# A joint penalized spline smoothing model for the number of positive and negative COVID-19 tests

**DOI:** 10.1371/journal.pone.0303254

**Published:** 2024-05-06

**Authors:** Dries De Witte, Ariel Alonso Abad, Thomas Neyens, Geert Verbeke, Geert Molenberghs

**Affiliations:** 1 L-BioStat, KU Leuven, Leuven, Belgium; 2 I-BioStat, UHasselt, Diepenbeek, Belgium; Marche Polytechnic University, ITALY

## Abstract

One of the key tools to understand and reduce the spread of the SARS-CoV-2 virus is testing. The total number of tests, the number of positive tests, the number of negative tests, and the positivity rate are interconnected indicators and vary with time. To better understand the relationship between these indicators, against the background of an evolving pandemic, the association between the number of positive tests and the number of negative tests is studied using a joint modeling approach. All countries in the European Union, Switzerland, the United Kingdom, and Norway are included in the analysis. We propose a joint penalized spline model in which the penalized spline is reparameterized as a linear mixed model. The model allows for flexible trajectories by smoothing the country-specific deviations from the overall penalized spline and accounts for heteroscedasticity by allowing the autocorrelation parameters and residual variances to vary among countries. The association between the number of positive tests and the number of negative tests is derived from the joint distribution for the random intercepts and slopes. The correlation between the random intercepts and the correlation between the random slopes were both positive. This suggests that, when countries increase their testing capacity, both the number of positive tests and negative tests will increase. A significant correlation was found between the random intercepts, but the correlation between the random slopes was not significant due to a wide credible interval.

## 1 Introduction

A new coronavirus, named COVID-19, was first detected in Wuhan, China in December 2019 [1]. This highly infectious virus has rapidly spread around the world and on March 11, 2020, the COVID-19 outbreak was officially declared a global pandemic by the World Health Organization (WHO). In response, countries have implemented sets of non-pharmaceutical interventions (e.g., face mask mandates, social distancing, stay-at-home orders, perimeters) to control the spread. Alongside these measures, testing and contact tracing are very important tools to fight the pandemic. By testing, we can identify infected individuals and isolate them to prevent further transmission [2]. An earlier study showed that increasing the number of tests helps to reduce the number of new COVID-19 cases [3]. Petrof et al. [4] studied the effect of doubling of cases on key metrics, with the emphasis on hospitalizations and mortality. Demographic, social, health risk, medical and environmental factors influencing the risk of testing positive or negative were studied by Chaeau-Hyam et al. [5].

Moreover, testing can help us better understand the spread of the pandemic by providing information on a variety of epidemiological indicators, such as the total number of tests conducted per day, the number of positive tests (confirmed cases), the number of negative tests and the positivity rate [6]. These indicators vary with time and are interconnected; the positivity rate can be obtained by dividing the number of positive tests by the total number of tests. The total number of tests can be calculated from the number of positive and negative tests. In this study, the aim is to better understand the complex relationship between these interconnected indicators by studying the association between the daily number of new positive tests (confirmed cases) and the daily number of new negative tests, as well as the time trend therein. In addition, when countries increased their testing capacity, the question raised by the general public was whether the increase in the number of daily cases would not merely be due to the increase in the number of daily tests; when more tests are carried out, more positive cases can be found. This paper aims to show that an increase in the daily number of positive tests is also associated with an increase in the daily number of negative tests. To this end, we analyzed data that were recorded daily for several countries.

To study the association between two longitudinal outcomes, a joint multivariate model is necessary. A review of the statistical approaches that have been proposed to model multivariate longitudinal data can be found in the paper of Verbeke et al. [7]. For the purpose of our study, we chose a random-effects approach for the joint modeling of multivariate longitudinal processes [8]. Since the longitudinal trajectories of the two outcomes of interest are nonlinear, we made use of spline methods. Spline functions are piecewise polynomial functions that are tied together. The values at which the piecewise functions are joined, are called knots. A spline function can be modeled as a linear combination of basis functions. In general, three types of spline approaches can be distinguished. The first approach is often referred to as the regression splines approach: a small number of knots is selected, together with the knot locations. The coefficients of the basis functions are estimated as in an ordinary regression model. However, an important drawback of regression splines is that results can be sensitive to the number and location of the knots. A spline method that alleviates this problem is the smoothing splines approach, where all distinct time points are used as knots. This obviates the need to select the number and location of the knots. The coefficients are estimated using penalization to avoid overfitting. Nevertheless, the computational burden increases with the number of time points, and thus the number of knots. This has been the main motivation for the development of so-called penalized splines. These can be seen as a hybrid between the first two spline methods. In this approach, a relatively large number of knots is chosen such that the influence of the choice of knots is minimized. But to reduce the computational burden of the smoothing spline approach, the number of knots is typically much smaller than the number of distinct time points. The coefficients are again estimated using a penalty to avoid overfitting [9, 10]. Many types of spline bases exist, such as a truncated power basis and a B-spline basis [11]. A B-spline basis is often preferred over other types of bases since it has better numerical properties. In this study, we considered P-splines smoothing, which is a specific type of penalized splines smoothing that uses a B-spline basis [12]. Models using penalized splines or P-splines can be represented as linear mixed models, making them very easy to implement with standard software [13, 14].

Although the use of penalized splines and P-splines for univariate longitudinal data is well-known, its use for multivariate longitudinal data seems to be limited. Zhao et al. [15] used penalized splines to jointly model the longitudinal trajectories of HIV viral load levels and CD4 counts. However, in their model, they only used random intercepts and slopes for the subject-specific trajectories, assuming that the individual trends are linear. We propose a model in which the subject-specific deviations from the common spline for the mean profile are modeled using nonparametric functions, allowing the subject-specific trajectories to be nonlinear [16–18]. To the best of our knowledge, this is the first study that has used this methodology in a joint model for multivariate longitudinal data.

Furthermore, serial correlation was taken into account by assuming first-order autoregressive errors for each country [10, 19]. We modeled heteroscedasticity by allowing the residual variances and autocorrelation parameters to be country-specific [20, 21], which at the same time accommodates differing country sizes.

The rest of the paper is organized as follows. The data used are described in Section 2. The methodology is laid out in Section 3, and the results of applying it to the data is the subject of Section 4.

## 2 Data

We obtained publicly available data from the website of Our world in Data (OWID) [22, 23]. The data set provided by OWID is updated daily and contains official COVID-19 data for several countries worldwide. The analysis was performed for all countries in the European Union (Austria, Belgium, Bulgaria, Croatia, Republic of Cyprus, Czech Republic, Denmark, Estonia, Finland, France, Germany, Greece, Hungary, Ireland, Italy, Latvia, Lithuania, Luxembourg, Malta, Netherlands, Poland, Portugal, Romania, Slovakia, Slovenia, Spain and Sweden), Switzerland, the United Kingdom and Norway.

To model the daily number of positive tests, we used the 7-day rolling average of new confirmed COVID-19 cases (positive tests) per 1,000,000 people. The 7-day rolling average at any given day (*t*) was calculated by taking the average of the COVID-19 confirmed cases over the previous 7 days, including the current day (*t*) and the preceding 6 days. Since the OWID data set does not contain information on the number of negative tests, we calculated the 7-day rolling average of new negative tests for COVID-19 per 1,000,000 people based on the 7-day rolling average of new confirmed COVID-19 cases per 1,000,000 people and the 7-day rolling average of new total tests for COVID-19 per 1,000 people. The daily COVID-19 test counts can exhibit considerable day-to-day variability due to various factors such as reporting delays, Monday, and weekend effects. By using a rolling average, the data is smoothed out over a 7-day period, reducing the impact of these short-term fluctuations and providing a clearer picture of the underlying trend. Nevertheless, as a sensitivity analysis, we also analyzed the daily number of new positive and negative tests per 1,000,000 people, while correcting for possible weekend effects.

We first performed the analysis for the period of March 2020 until June 23, 2022, since the Our World in Data (OWID) dataset only included data until that date. However, since the Omicron variant had unique characteristics and transmission patterns, it could have influenced the outcomes we were investigating in ways that differ from previous waves. Therefore, we also performed the analysis for the period of March 2020 until November 2021, excluding the Omicron wave, and for the period of November 2021 until June 2022, including only the Omicron wave. This was done to see if the Omicron wave altered the results. Since we analyzed the 7-day rolling average instead of the daily number of new positive and negative tests, which are count data, we considered the two variables as continuous responses. As both responses are positive, we log-transformed both variables. Even though the raw data are, strictly speaking, counts, models for Gaussian data were used to analyze the data instead of a Poisson model, since Gaussian models allow us to model the mean and variance parameters separately while at the same time providing a great deal of flexibility. Considering this, together with the oftentimes very large observed values, we opted to employ models for Gaussian data. The log-transformation is then a natural choice.

## 3 Methodology

The aim of our analysis was to model the two responses of interest, the log-transformed 7-day rolling average number of new positive tests and the the log-transformed 7-day rolling average number of new negative tests, simultaneously. For this purpose, we used the random-effects approach for the joint modeling of multivariate longitudinal processes [8]. In this approach, separate linear mixed models with random effects are assumed for each outcome. The two outcomes are then linked to each other by imposing a joint multivariate distribution on the random effects.

First, a model for each of the two outcomes was built. Since the longitudinal trajectories of both responses are nonlinear, we made use of P-splines [10, 12, 24]. A general nonparametric model using splines can be formulated as

$$\begin{array}{c} y_{i}=f\left(x_{i}\right)+\epsilon_{i},\;\epsilon_{i}∼N\left(0,\sigma_{\epsilon }^{2}\right), \end{array}$$ (1)

with *y*_*i*_ the vector of responses and *f*(*x*_*i*_) the smooth function of a covariate *x*. We can express this smooth function *f*(⋅) as a linear combination of *d* B-spline basis functions *B*_*j*_:

$$\begin{array}{c} f\left(x_{i}\right)=\sum_{j=1}^{d}B_{j}\left(x_{i}\right)\theta_{j}, \end{array}$$ (2)

where *θ*_*j*_ is the unknown spline coefficient associated with basis function *B*_*j*_. When using a penalized spline smoothing approach, a relatively large number of knots is chosen. Ruppert et al. [10] advised to select the number of knots by using a rule, i.e., min(*numberofuniquevaluesofx*/4, 40). Following this rule, 40 equally-spaced knots were selected. Overfitting is accounted for by imposing a penalty. Eilers et al. [12] proposed to use a difference penalty on the coefficients of adjacent B-splines. Let *θ* be the vector of unknown spline coefficients *θ*_*j*_ and *B* the matrix of B-spline basis functions, then *θ* can be estimated by minimizing the penalized sum of squares:

$$\begin{array}{c} PSS={\left(y-B\theta \right)}^{\prime }\left(y-B\theta \right)+\lambda \theta^{\prime }P\theta , \end{array}$$ (3)

where *P* represents the penalty matrix. The amount of smoothness is controlled by introducing a smoothing parameter λ. *P* can then be expressed as *P* = *D*′*D*, with *D* equal to a difference matrix of order *q*. Typically, a second-order penalty is chosen (*q* = 2).

Models using P-splines are often reparameterized as mixed-effects models of the form *y* = *Xβ* + *Zu* + *ϵ* [24]. One major benefit of this is that it allows us to fit the models using standard fitting tools for mixed models. To express the P-Spline smoothing model as a linear mixed model, a transformation of the model’s B-spline basis *B* into a new model basis [*X*: *Z*] is needed, such that *y* = *Xβ* + *Zu* + *ϵ*. This representation can be seen as a decomposition of the fitted values, *y*, into an unpenalized part, *Xβ* and a penalized part, *Zu*. The approach that we followed is that suggested by Currie et al. [25] and Lee [26], which is based on the singular value decomposition of the penalty matrix *P*:

$$\begin{array}{c} D^{\prime }D=U\Sigma U^{\prime }, \end{array}$$ (4)

with *U* the matrix containing the eigenvectors and Σ a diagonal matrix containing the eigenvalues, with the number of null eigenvalues equal to *q*. The matrix *U* can be partitioned into two parts:

$$\begin{array}{c} U=[U_{n}:U_{s}], \end{array}$$ (5)

where *U*_*n*_ is the matrix of eigenvectors that correspond to the null eigenvalues and *U*_*s*_ is the matrix of eigenvectors corresponding to the non-zero eigenvalues. Hence, the penalty *P* can be decomposed as

$$\begin{array}{c} D^{\prime }D=\left[U_{n}:U_{s}\right][\begin{array}{cc} 0_{q} & 0\\ 0 & \tilde{\Sigma } \end{array}][\begin{array}{c} U_{n}^{\prime }\\ U_{s}^{\prime } \end{array}], \end{array}$$ (6)

with $\tilde{\Sigma }$ a diagonal matrix that contains the positive eigenvalues of Σ. 0_*q*_ is a square matrix of zeros. Since a second-order penalty was chosen, i.e., *q* = 2, there are two eigenvalues equal to zero in Σ and *U*_*n*_ has two columns [27]. The goal is to find a transformation matrix *T*, such that *BT* = [*X*: *Z*]. Following Lee [26], we define *T* as an orthogonal matrix *T* = [*U*_*n*_: *U*_*s*_]. Using this transformation, we can define the fixed and random effects matrices as

$$\begin{array}{c} \begin{array}{ccc} X & = & BU_{n},\\ Z & = & BU_{s}. \end{array} \end{array}$$ (7)

The corresponding coefficients then become

$$\begin{array}{c} \begin{array}{ccc} \beta & = & U_{n}^{\prime }\theta ,\\ u & = & U_{s}^{\prime }\theta , \end{array} \end{array}$$ (8)

which results in the linear mixed model

$$\begin{array}{c} \begin{array}{ccc} y & = & X\beta +Zu+\epsilon ,\\ u & ∼ & N\left(0,G\right),\epsilon ∼N\left(0,\sigma^{2}\right), \end{array} \end{array}$$ (9)

with *G*, the variance components matrix, equal to $\sigma_{u}^{2}{\tilde{\Sigma }}^{-1}$. It can be shown that the smoothing parameter λ then becomes $\sigma^{2}/\sigma_{u}^{2}$. According to Lee [26], the fixed effects matrix *X* = *BU*_*n*_ can be replaced by any sub-matrix such that the composed matrix [*X*: *Z*] has full rank and *X* and *Z* are orthogonal. Since we choose a second order penalty, the fixed effects matrix *X* can be taken as a matrix with two columns; a column of ones, and a column containing the covariate vector *x*, *X* = [1: *x*]. Additionally, by defining the random effects matrix *Z* as $BU_{s}{\tilde{\Sigma }}^{-0.5}$, it can be shown that *G* is equal to $\sigma_{u}^{2}I$ [26, 27].

The mixed-effects model representation is especially useful for analyzing longitudinal data, as this allows a straightforward extension of the model by including additional random effects [9, 14]. The most flexible model is one in which the subject-specific differences are allowed to be nonparameteric functions as well [16]. Let *y*_*ij*,*l*_ denote the response of interest, either the log-transformed 7-day rolling average number of new positive tests (*l* = 1) or the log-transformed 7-day rolling average number of new negative tests (*l* = 2), for country *i*, measured at day *t*_*ij*_, *i* = 1, …, *N*, *j* = 1, …, *n*_*i*_. The model using P-splines smoothing, represented as a linear mixed model, in which the country-specific trends are also smoothed is:

$$\begin{array}{ccc} y_{ij,l} & = & f_{l}(t_{ij})+g_{i,l}(t_{ij})+\epsilon_{ij,l},\\ & & \epsilon_{ij,l}\sim N(0,\sigma_{\epsilon_{l}}^{2}),\\ f_{l}(t_{ij}) & = & \beta_{0,l}+\beta_{1,l}t_{ij,l}+\sum_{k=1}^{K}\\ & & b_{k,l}z_{k},b_{k,l}\sim N(0,\sigma_{b,l}^{2}),\\ g_{i,l}(t_{ij,l}) & = & \delta_{0i,l}+\delta_{1i,l}t_{ij}+\sum_{k=1}^{K}e_{k}z_{k}+\sum_{k=1}^{K}d_{ki,l}z_{k},\\ & & {\left(\delta_{0i,l},\delta_{1i,l}\right)}^{T}\sim N(0,D)\;d_{ki,l}\sim N(0,\sigma_{d,l}^{2}), \end{array}$$ (10)

where *g*_*i*,*l*_(*x*_*ij*_) is the country-specific penalized spline smoothing the country-specific deviations from the overall penalized spline *f*_*l*_(*x*_*ij*_). *δ*_0*i*,*l*_ and *δ*_1*i*,*l*_ are the random intercepts and slopes, respectively, representing the linear part of the model. The nonlinear component is represented by $\sum_{k=1}^{K}d_{ki,l}z_{k}$. This country-specific penalized spline component of the model can account for deviations from the country-specific linear trend.

In model (10), the error terms are assumed to be independent and identically normally distributed with mean zero and residual variance $\sigma_{\epsilon_{l}}^{2}$. However, since the data are collected daily, it is likely that this assumption is not valid and the residuals are serially correlated. To take into account the autocorrelation between the error terms *ϵ*_*ij*,*l*_, we implemented an autoregressive model of order one (AR(1)) for the errors [19, 28]. Let *ϵ*_*ij*,*l*_ be the error term for country *i* at timepoint *j* for outcome *l*. Then,

$$\begin{array}{c} \epsilon_{ij,l}=\rho_{l}\epsilon_{ij-1,l}+\omega_{ij,l}, \end{array}$$ (11)

with *ρ*_*l*_ the autocorrelation parameter. Since it is assumed that *ρ*_*l*_ is a correlation parameter, we have |*ρ*_*l*_| < 1. Furthermore, *ω*_*ij*,*l*_ is the new error term which is assumed to be independent and identically normally distributed with mean zero and variance $\sigma_{l}^{2}$. We also assume stationarity. This implies that

$$\begin{array}{c} \begin{array}{ccc} \text{Var}(\epsilon_{ij,l}) & = & \frac{\sigma_{l}^{2}}{1-\rho_{l}^{2}},\\ \text{Cov}(\epsilon_{ij,l},\epsilon_{ij-1,l}) & = & \rho_{l}(\frac{\sigma_{l}^{2}}{1-\rho_{l}^{2}}). \end{array} \end{array}$$ (12)

Furthermore, to account for heteroscedasticity, we allowed the autocorrelation parameters and residual variances to vary among countries. By incorporating country-specific residual variances and autocorrelation parameters, along with country-specific penalized splines and random effects, our model becomes very flexible so that it can accommodate potential sources of differential measurement error. Nevertheless, the residuals are still assumed homoscedastic within a country. Log-transformed country-specific residual variances and -log(-log())-transformed country-specific autocorrelation parameters were modeled as random effects resulting from a normal distribution. The -log(-log())-transformation for the country-specific autocorrelation parameters was applied since the autocorrelation parameters are bounded by −1 and +1 and the log-transformation for the country-specific residual standard deviations was chosen since variances are always positive [20, 21, 29]. Let *y*_*ij*,*l*_ again denote the response of interest as described before. Then the models outlined in (10) can be extended as:

$$\begin{array}{c} \begin{array}{ccc} y_{ij,l} & = & f_{l}\left(t_{ij}\right)+g_{i,l}\left(t_{ij}\right)+\epsilon_{ij,l},\\ \epsilon_{ij,l} & = & \rho_{i,l}\epsilon_{ij-1,l}+\omega_{ij,l},\\ \omega_{ij,l} & ∼ & N(0,\sigma_{i,l}^{2}),\\ \text{log}(\sigma_{i,l}) & ∼ & N(\mu_{\omega_{l}},\sigma_{\omega_{i,l}}^{2}),\\ -\text{log}(-\text{log}\left(\rho_{i,l}\right)) & ∼ & N(\mu_{\rho_{l}},\sigma_{\rho_{i,l}}^{2}). \end{array} \end{array}$$ (13)

To link these two linear mixed models to each other, a joint 4-dimensional multivariate Normal distribution with zero mean and 4 × 4-dimensional variance-covariance matrix is imposed on the random intercepts and slopes. Assuming that the association between the two outcomes can be fully captured by the association between the random intercepts and slopes, we can derive the association from the correlation between these random effects. Let *y*_*ij*,*p*_ be the log-transformed 7-day rolling average number of new positive tests for country *i* measured at day *t*_*ij*_ and let *y*_*ij*,*n*_ be the log-transformed 7-day rolling average number of new negative tests, for country *i*, measured at day *t*_*ij*_, *i* = 1, …, *N*, *j* = 1, …, *n*_*i*_. Then the final joint model that we fitted for the two responses is:

$$\begin{array}{c} \begin{array}{ccc} y_{ij,p}\;\; & \;\;=\;\; & \;\;f_{p}\left(t_{ij}\right)+g_{i,p}\left(t_{ij}\right)+\epsilon_{ij,p},\\ \epsilon_{ij,pv}\;\; & \;\;=\;\; & \;\;\rho_{i,p}\epsilon_{ij-1,p}+\omega_{ij,p},\\ \omega_{ij,p}\;\; & \;\;∼\;\; & \;\;N(0,\sigma_{i,p}^{2}),\\ \text{log}(\sigma_{i,p}\;\;) & \;\;∼\;\; & \;\;N(\mu_{\omega_{p}},\sigma_{\omega_{i,p}}^{2}),\\ -\text{log}(-\text{log}\left(\rho_{i,p}\right))\;\; & \;\;∼\;\; & \;\;N(\mu_{\rho_{p}},\sigma_{\rho_{i,p}}^{2}),\\ f_{p}\left(t_{ij}\right)\;\; & \;\;=\;\; & \;\;\beta_{0,p}+\beta_{1,p}t_{ij}+\sum_{k=1}^{K}b_{k,p}z_{k},\\ b_{k,p}\;\; & \;\;∼\;\; & \;\;N(0,\sigma_{b,p}^{2}),\\ g_{i,p}\left(t_{ij}\right)\;\; & \;\;=\;\; & \;\;\delta_{0i,p}+\delta_{1i,p}t_{ij}+\sum_{k=1}^{K}d_{ki,p}z_{k},\\ d_{ki,p}\;\; & \;\;∼\;\; & \;\;N(0,\sigma_{d,p}^{2}),\\ y_{ij,n}\;\; & \;\;=\;\; & \;\;f_{n}\left(t_{ij}\right)+g_{i,n}\left(t_{ij}\right)+\epsilon_{ij,n},\\ \epsilon_{ij,n}\;\; & \;\;=\;\; & \;\;\rho_{i,p}\epsilon_{ij-1,n}+\omega_{ij,n},\\ \omega_{ij,n}\;\; & \;\;∼\;\; & \;\;N(0,\sigma_{i,n}^{2}),\\ \text{log}(\sigma_{i,n})\;\; & \;\;∼\;\; & \;\;N(\mu_{\omega_{n}},\sigma_{\omega_{i,n}}^{2}),\\ -\text{log}(-\text{log}\left(\rho_{i,n}\right))\;\; & \;\;∼\;\; & \;\;N(\mu_{\rho_{n}},\sigma_{\rho_{i,n}}^{2}),\\ f_{n}\left(t_{ij}\right)\;\; & \;\;=\;\; & \;\;\beta_{0,n}+\beta_{1,n}t_{ij}+\sum_{k=1}^{K}b_{k,n}z_{k},\\ b_{k,n}\;\; & \;\;∼\;\; & \;\;N(0,\sigma_{b,n}^{2})\\ g_{i,n}\left(t_{ij}\right)\;\; & \;\;=\;\; & \;\;\delta_{0i,n}+\delta_{1i,n}t_{ij}+\sum_{k=1}^{K}d_{ki,n}z_{k},\\ d_{ki,n}\;\; & \;\;∼\;\; & \;\;N(0,\sigma_{d,n}^{2}), \end{array} \end{array}$$ (14)

$$\begin{array}{c} {\left(\delta_{0i,p},\delta_{1i,p},\delta_{0i,n},\delta_{1i,n}\right)}^{T}∼N\left(0,D\right), \end{array}$$

with *D* the variance-covariance matrix of the random intercepts and slopes from which we can obtain the correlations of interest. We did not induce correlation between the non-linear components of the model, since introducing correlation between the non-linear components might add complexity to the model while making the interpretation more convoluted. The non-linear components involve complex structures so that the correlation can be challenging to interpret in a straightforward manner, since the smoothing random effects used in the non-linear components only serve as a numerical and computational trick to fit the penalized splines efficiently. Hence, the random effects used to model the penalized splines are not of the same nature as the random intercepts and random slopes.

Based on the correlations between the random intercepts and slopes, we can obtain the marginal correlation function between both outcomes as a function of time [8]:

$$\begin{array}{c} r_{M}\left(t\right)=\frac{\sigma_{\delta_{0i,p},\delta_{0i,n}}+t\sigma_{\delta_{0i,p},\delta_{1i,n}}+t\sigma_{\delta_{0i,n},\delta_{1i,p}}+t^{2}\sigma_{\delta_{1i,p},\delta_{1i,n}}}{\sqrt{\sigma_{\delta_{0i,p}}^{2}+2t\sigma_{\delta_{0i,p},\delta_{1i,p}}+t^{2}\sigma_{\delta_{1i,p}}^{2}+\sigma_{p}^{2}}\sqrt{\sigma_{\delta_{0i,n}}^{2}+2t\sigma_{\delta_{0i,n},\delta_{1i,n}}+t^{2}\sigma_{\delta_{1i,n}}^{2}+\sigma_{n}^{2}}} \end{array}$$ (15)

### 3.1 Model implementation and fitting

Bayesian methods were used to estimate the model parameters. The posterior distributions were approximated using Markov Chain Monte Carlo (MCMC) methods. Therefore, Gibbs sampling was used as MCMC algorithm. The model was implemented in JAGS [30], version 4.3.1, using the runjags v.2.2.1 package [31] in R v.4.2.0 [32].

To improve convergence, we hierarchically centered the random intercepts and slopes. Posterior correlation among the population-level intercepts and slopes was reduced by mean centering the time variable *t* [33]. We ran four parallel chains with random generated starting values for 300,000 iterations each. The first 200,000 iterations of each chain were discarded as burn-in period. To summarize the remaining posterior samples, we report the posterior means, standard deviations and 95% equal-tailed credible intervals. Convergence was assessed by looking at the traceplots, effective sample size (ESS) and the Potential Scale Reduction Factor (PSRF) [34]. All parameters had a PSRF value less than 1.05, indicating that convergence was acceptable [35].

#### 3.3.1 Priors

We chose weakly informative prior distributions [36]. For the *β* parameters, independent normal distributions with mean zero and a variance of 10,000 were used. For the variance parameters of the normal distributions of the penalized coefficients ($\sigma_{b,p}^{2},\sigma_{d,p}^{2},\sigma_{b,n}^{2},\sigma_{d,n}^{2}$), the variances of the normal distributions of the country-specific autocorrelation parameters ($\sigma_{\rho ,p}^{2},\sigma_{\rho ,n}^{2}$), and the variances of the normal distributions of the country-specific residual variances ($\sigma_{\omega_{p}}^{2},\sigma_{\omega_{n}}^{2}$), we chose Inverse-Gamma distributions with the shape and scale parameters equal to 0.001. Normal distributions with mean equal to 0 and variance equal to 100 were specified for the mean parameters of the normal distributions of the -log(-log)-transformed country-specific autocorrelation parameters ($\mu_{\rho_{p}},\mu_{\rho_{n}}$) and for the mean parameters of the normal distributions of the log-transformed country-specific residual variances ($\mu_{\omega_{p}},\mu_{\omega_{n}}$). In summary, the priors implemented were:

$$\begin{array}{c} \begin{array}{ccc} \beta_{0,p},\beta_{1,p},\beta_{0,n},\beta_{1,n} & ∼ & N(0,10000),\\ \sigma_{b,p}^{2},\sigma_{d,p}^{2},\sigma_{b,n}^{2},\sigma_{d,n}^{2} & ∼ & IG(0.001,0.001),\\ \sigma_{\omega_{p}}^{2},\sigma_{\omega_{n}}^{2} & ∼ & IG(0.001,0.001),\\ \sigma_{\rho_{p}}^{2},\sigma_{\rho_{n}}^{2} & ∼ & IG(0.001,0.001),\\ \mu_{\rho_{p}},\mu_{\rho_{n}} & ∼ & N(0,100),\\ \mu_{\omega_{p}},\mu_{\omega_{n}} & ∼ & N(0,100). \end{array} \end{array}$$ (16)

Instead of the commonly used Inverse-Wishart prior, a hierarchical Inverse-Wishart prior was used for the variance-covariance matrix of the random intercepts and slopes, *D*, as suggested by Huang and Wand [37]. They proposed an Inverse-Wishart (IW) distribution as prior for *D*, with Inverse-Gamma (IG) prior distributions for the diagonal elements of the scale matrix.

$$\begin{array}{c} \begin{array}{ccc} D|d_{1},\ldots ,d_{4} & ∼ & IW(v+4-1,2v\text{diag}\left(1/d_{1},\ldots ,1/d_{4}\right)),\\ d_{k} & ∼ & IG(1/2,1/A_{k}^{2}),\;k=1,\ldots ,4. \end{array} \end{array}$$ (17)

This prior produces half-*t*(*v*, *A*_*k*_) distributions on the standard deviations of *D*. By specifying large values of $A_{k}^{2}$, e.g., 10^3^, weakly informative priors are implied on the standard deviations [38]. We set *v* equal to 2 as this leads to marginal uniform distributions for the correlation parameters. As shown in the paper by Ariyo et al. [39], the hierarchical Inverse-Wishart prior for the variance-covariance matrix of the random effects, combined with Inverse-Gamma priors for the other variance parameters, outperforms the classical Inverse-Wishart prior in linear mixed models.

### 3.2 Sensitivity analyses

#### 3.2.1 Priors

The impact of the prior distributions was examined through a sensitivity analysis. The scale and shape parameters of the Inverse-Gamma prior distributions for the variance parameters were changed to 0.01 and a variance of 1000 was chosen for the normal priors for the *β*-coefficients. We also set $A_{k}^{2}$ equal to 10^2^ to examine the impact of the chosen prior for the variance-covariance matrix *D*. The impact of the prior distributions for the mean parameters of the normal distributions of the log-transformed country-specific residuals variances and the -log(-log)-transformed country-specific autocorrelation parameters was examined by decreasing the variance from 100 to 10. All prior distributions then become:

$$\begin{array}{c} \begin{array}{ccc} \beta_{0,p},\beta_{1,p},\beta_{0,n},\beta_{1,n} & ∼ & N(0,1000),\\ \sigma_{b,p}^{2},\sigma_{d,p}^{2},\sigma_{b,n}^{2},\sigma_{d,n}^{2} & ∼ & IG(0.01,0.01),\\ \sigma_{\omega_{p}}^{2},\sigma_{\omega_{n}}^{2} & ∼ & IG(0.01,0.01),\\ \sigma_{\rho_{p}}^{2},\sigma_{\rho_{n}}^{2} & ∼ & IG(0.01,0.01),\\ \mu_{\rho_{p}},\mu_{\rho_{n}} & ∼ & N(0,10),\\ \mu_{\omega_{p}},\mu_{\omega_{n}} & ∼ & N(0,10), \end{array} \end{array}$$ (18)

and

$$\begin{array}{c} \begin{array}{ccc} D|d_{1},\ldots ,d_{4} & ∼ & IW(v+4-1,2v\text{diag}\left(1/d_{1},\ldots ,1/d_{4}\right)),\\ d_{k} & ∼ & IG(1/2,1/10^{2}),\;k=1,\ldots ,4. \end{array} \end{array}$$

#### 3.2.2 Daily number of positive and negative tests

As stated earlier, we also analyzed the daily number of new positive and new negative tests per 1,000,000 instead of the 7-day rolling average. To take into account possible week and weekend effects, an indicator variable was added as a covariate to the model. The fitted joint model then takes the form:

$$\begin{array}{c} \begin{array}{ccc} y_{ij,p}\;\; & \;\;=\;\; & \;\;f_{p}\left(t_{ij}\right)+g_{i,p}\left(t_{ij}\right)+\beta_{2,p}week_{i}+\epsilon_{ij,p},\\ \epsilon_{ij,p}\;\; & \;\;∼\;\; & \;\;N(0,\sigma_{i,p}^{2}),\\ \text{log}(\sigma_{i,p}\;\;) & \;\;∼\;\; & \;\;N(\mu_{\omega_{p}},\sigma_{\omega_{i,p}}^{2}),\\ f_{p}\left(t_{ij}\right)\;\; & \;\;=\;\; & \;\;\beta_{0,p}+\beta_{1,p}t_{ij}+\sum_{k=1}^{K}b_{k,p}z_{k},\\ b_{k,p}\;\; & \;\;∼\;\; & \;\;N(0,\sigma_{b,p}^{2}),\\ g_{i,p}\left(t_{ij}\right)\;\; & \;\;=\;\; & \;\;\delta_{0i,p}+\delta_{1i,p}t_{ij}+\sum_{k=1}^{K}d_{ki,p}z_{k},\\ d_{ki,p}\;\; & \;\;∼\;\; & \;\;N(0,\sigma_{d,p}^{2}),\\ y_{ij,n}\;\; & \;\;=\;\; & \;\;f_{n}\left(t_{ij}\right)+g_{i,n}\left(t_{ij}\right)+\beta_{2,n}week_{i}+\epsilon_{ij,n},\\ \epsilon_{ij,n}\;\; & \;\;∼\;\; & \;\;N(0,\sigma_{i,n}^{2}),\\ \text{log}(\sigma_{i,n})\;\; & \;\;∼\;\; & \;\;N(\mu_{\omega_{n}},\sigma_{\omega_{i,n}}^{2}),\\ f_{n}\left(t_{ij}\right)\;\; & \;\;=\;\; & \;\;\beta_{0,n}+\beta_{1,n}t_{ij}+\sum_{k=1}^{K}b_{k,n}z_{k},\\ b_{k,n}\;\; & \;\;∼\;\; & \;\;N(0,\sigma_{b,n}^{2})\\ g_{i,n}\left(t_{ij}\right)\;\; & \;\;=\;\; & \;\;\delta_{0i,n}+\delta_{1i,n}t_{ij}+\sum_{k=1}^{K}d_{ki,n}z_{k},\\ d_{ki,n}\;\; & \;\;∼\;\; & \;\;N(0,\sigma_{d,n}^{2}), \end{array} \end{array}$$ (19)

$$\begin{array}{c} {\left(\delta_{0i,p},\delta_{1i,p},\delta_{0i,n},\delta_{1i,n}\right)}^{T}∼N\left(0,D\right) \end{array}$$

## 4 Results

### 4.1 Exploratory data analysis

**Fig 1:**
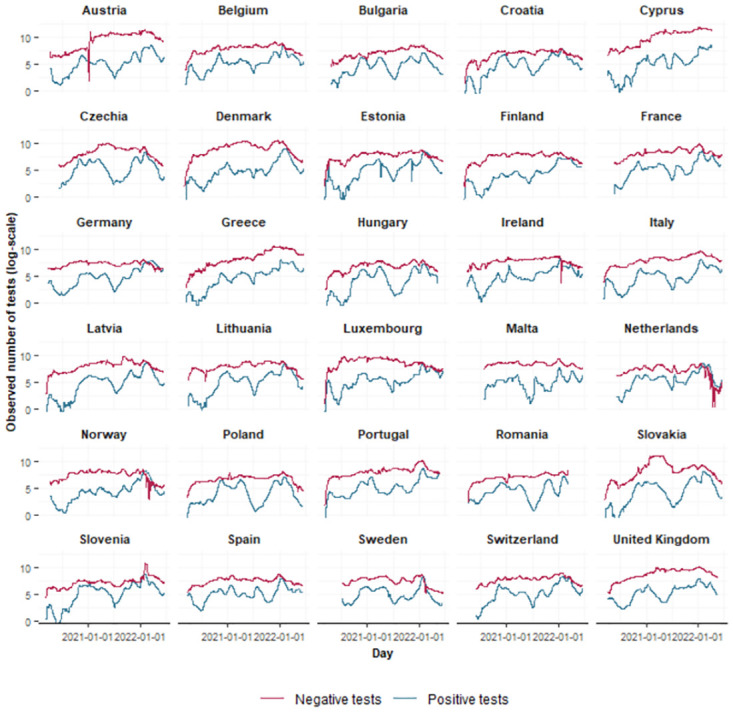
Observed trajectories. The observed log-transformed 7-day rolling average number of positive (blue) and negative (red) tests for each country.

Before fitting the model, we explored the 7-day rolling average number of new negative and positive COVID-19 tests per 1,000,000 people by means of summary statistics and graphical displays. The 7-day rolling average number of positive tests per 1,000,000 people across all countries ranges from 0.0 to 7833.4 with a mean value of 294.0 and a median value of 75.2. The mean of the 7-day rolling average number of negative tests per 1,000,000 people across all countries is equal to 6007.3, with a minimum and maximum value of 0 and 143501.8 respectively. The median is 2413.7. The mean, median, minimum and maximum 7-day rolling average number of positive and negative tests per 1,000,000 people for each country separately are reported in S1 and S2 Tables in the Supplemental Information. The observed country-specific trajectories of the 7-day rolling average number of positive and negative tests per 1,000,000 people are plotted in S1 and S2 Figs, respectively. Fig 1 shows the observed trajectories after log-transformation. There seems to be a lot of variation in the shape of the curves between countries, indicating that a sufficiently flexible model is needed.

### 4.2 Results of the joint model

After exploring the data, we fitted the joint model as described in (14). The correlations between the random effects, derived from the estimated variance-covariance matrix *D*, are given in Table 1 together with the corresponding 95% credible intervals. To study the relation between the number of positive tests and the number of negative tests, we are most interested in the correlation between the two random intercepts and the correlation between the two random slopes. The correlation between the random intercepts, *δ*_0*i*,*p*_ and *δ*_0*i*, *n*_, was equal to 0.46. The correlation between the random slopes, *δ*_1*i*,*p*_ and *δ*_1*i*,*n*_, was estimated as 0.21. These two positive correlations indicate that the number of positive tests is positively associated with the number of negative tests, hence, when the number of positive tests increases, there is also an increase in the number of negative tests. However, the correlation between the two random slopes was not significant as the 95% credible interval is quite wide and includes zero. The correlation between the random intercepts is significant.

**Table 1:** Correlations between the random intercepts and slopes.

	*δ*_0*i*,*p*_	*δ*_1*i*,*p*_	*δ*_0*i*,*n*_	*δ*_1*i*,*n*_
*δ*_0*i*,*p*_	1			
*δ*_1*i*,*p*_	0.24 [-0.31; 0.74]	1		
*δ*_0*i*,*n*_	0.46 [0.12; 0.77]	0.18 [-0.21; 0.55]	1	
*δ*_1*i*,*n*_	0.09 [-0.32; 0.48]	0.21 [-0.21; 0.61]	0.65 [0.37; 0.90]	1

**Fig 2:**
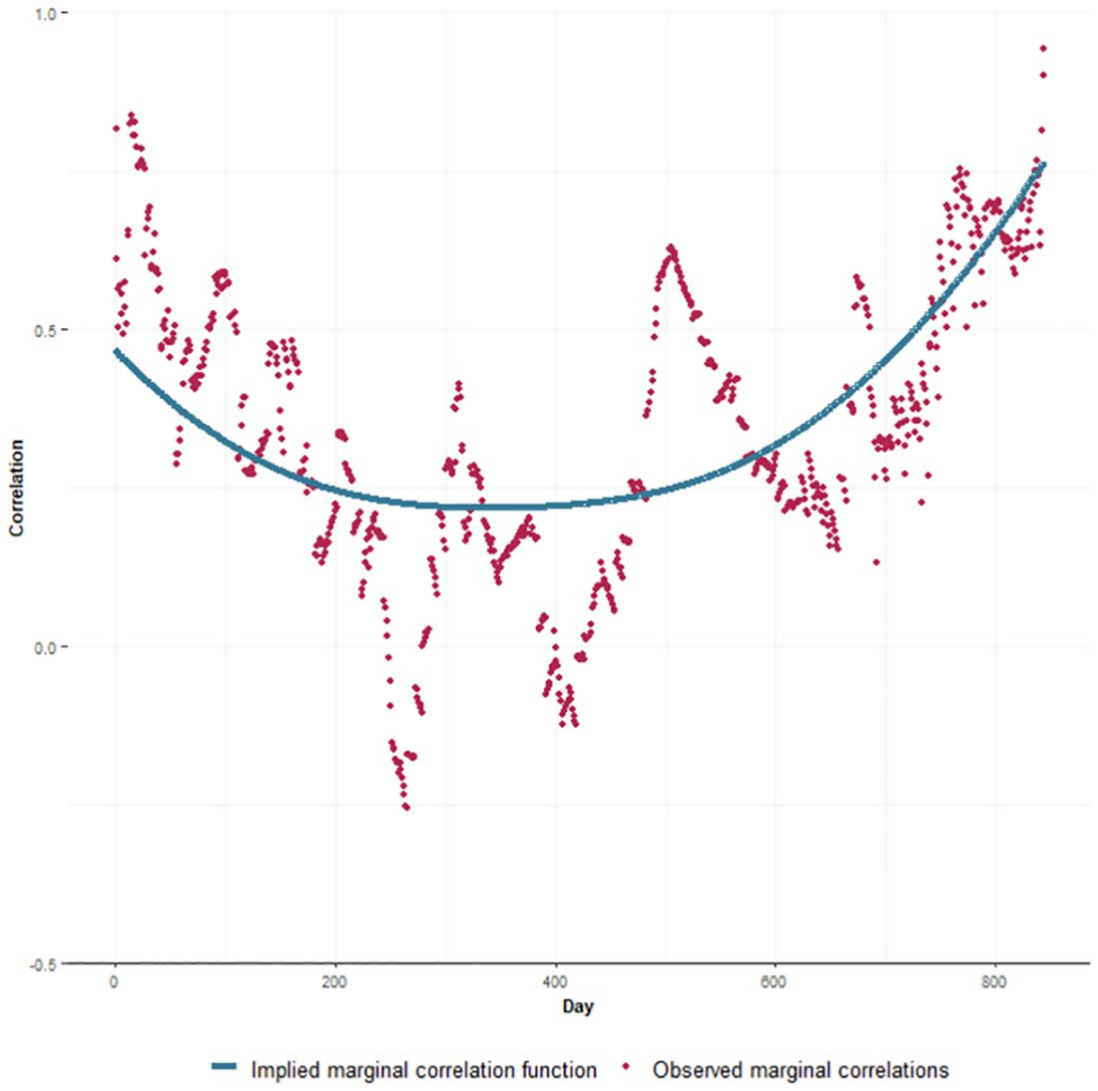
Marginal correlation function. Comparison of observed (dots) and implied (solid line) marginal correlation.

Fig 2 plots the marginal correlation function implied by the fitted joint model compared with the observed marginal correlations. These observed marginal correlations are calculated based on OLS residuals, obtained by subtracting the fixed effects and splines from the observed outcome values. Over time, there appears to be an initial slight decrease followed by an increase in both the implied marginal correlation function and the observed marginal correlations. It can also be seen that the form of the implied marginal correlation function seems to be compatible with the observed marginal correlations.

The parameter estimates for the fitted joint model are presented in Table 2. The estimated country-specific autocorrelation parameters and residual variances can be found in S3 Table. The country-specific autocorrelation parameters were very high for all countries, indicating that the autoregressive structure was indeed necessary. However, for Romania (*ρ*_*p*_ = 0.60), Poland (*ρ*_*p*_ = 0.63) and Latvia (*ρ*_*p*_ = 0.70), we noticed a substantially smaller autocorrelation parameter for the number of positive tests compared to other countries. For the number of negative tests, we noticed a smaller autocorrelation parameter for The Netherlands (*ρ*_*n*_ = 0.50) and Norway (*ρ*_*n*_ = 0.58). These variations in the autocorrelation parameters suggest that the dynamics of the number of tests may differ from country to country, and may indicate that using country-specific autocorrelation parameters instead of using one fixed parameter is necessary. The low autocorrelation parameters for certain countries could indicate the presence of differences in the trends among the regions within a country. This can also be the reason why larger residuals for these particular countries are observed (Figs 4 and 5). Looking at the residual variances, the estimated country-specific residual variance for the number of negative tests for Austria ($\sigma_{\omega_{n}}^{2}=0.05$) was very high. For the number of positive tests, the estimated country-specific residual variance for Estonia ($\sigma_{\omega_{n}}^{2}=0.11$) was very high. A graphical representation of these subject-specific parameters can be found in S3 Fig.

**Table 2:** Parameter estimates of the joint model.

	Mean	SD	95%CI
Submodel for the number of positive tests			
*β*_0,*p*_	4.3894	0.1321	[4.1427; 4.6642]
*β*_1,*p*_	0.0077	0.0004	[0.0068; 0.0087]
$\sigma_{b,p}^{2}$	2.4050	0.6493	[1.4396; 3.9515]
$\sigma_{d,p}^{2}$	0.6521	0.1006	[0.4565; 0.8442]
$\mu_{\omega_{p}}$	-1.9310	0.0593	[-2.0464; -1.8126]
$\sigma_{\omega_{p}}^{2}$	0.0975	0.0264	[0.0636; 0.1964]
$\mu_{\rho_{p}}$	2.4899	0.3779	[1.8155; 3.2879]
$\sigma_{\rho_{p}}^{2}$	2.2024	1.1546	[1.0547; 8.4105]
Submodel for the number of negative tests			
*β*_0,*n*_	7.4283	0.1958	[7.0430; 7.8176]
*β*_1,*n*_	0.0038	0.0004	[0.0029; 0.0046]
$\sigma_{b,n}^{2}$	1.3144	0.3837	[0.7398; 2.2267]
$\sigma_{d,n}^{2}$	0.1676	0.0262	[0.1201; 0.2223]
$\mu_{\omega_{n}}$	-2.6637	0.1130	[-2.8770; -2.4321]
$\sigma_{\omega_{n}}^{2}$	0.3529	0.0947	[0.2310; 0.7037]
$\mu_{\rho_{n}}$	4.3134	0.5888	[3.1321; 5.4615]
$\sigma_{\rho_{n}}^{2}$	7.1736	3.1004	[3.8491; 27.2479]

To assess the benefit of using country-specific residual variances and autocorrelation parameters, we compared the model with country-specific residual variances and autocorrelation parameters (full model) to two reduced models. In the first reduced model, we simplified the modeling of residual variances by using two fixed parameters: one for the number of positive tests and one for the number of negative tests, instead of modeling them as country-specific random effects. To evaluate the model fit, we calculated the Deviance Information Criterion (DIC) for each model, with lower DIC values indicating better model fit. The DIC for this reduced model (-96318) is slightly higher than the DIC of the full model (DIC: -97130), which indicates that accounting for country-specific variability in residual variances improved the model fit compared to a simpler approach. In the second reduced model, we simplified the modeling of autocorrelation by using two fixed parameters, rather than modeling them as country-specific random effects. For this model, the DIC was -77607, again indicating that this simpler model gives a worse fit than the full model.

**Fig 3:**
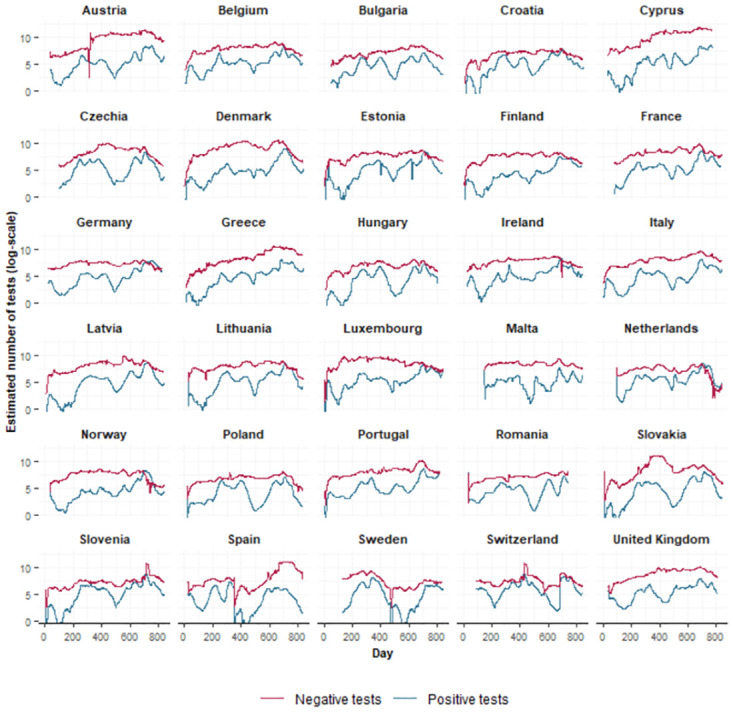
Estimated trajectories. The estimated log-transformed 7-day rolling average number of positive (blue) and negative (red) tests for each country.

The estimated longitudinal trajectories are plotted in Fig 3. The estimated longitudinal trajectories closely agree with the observed longitudinal trajectories, indicating that the joint model fits the data well. To illustrate this further, a figure of the estimated longitudinal trajectories together with the observed longitudinal trajectories (S4 Fig) is included in the Supplemental Information. From the estimated number of positive and negative tests obtained from our model, we can also derive the positivity rate. In S5 Fig in the Supplemental Information, the calculated positivity rate is shown.

**Fig 4:**
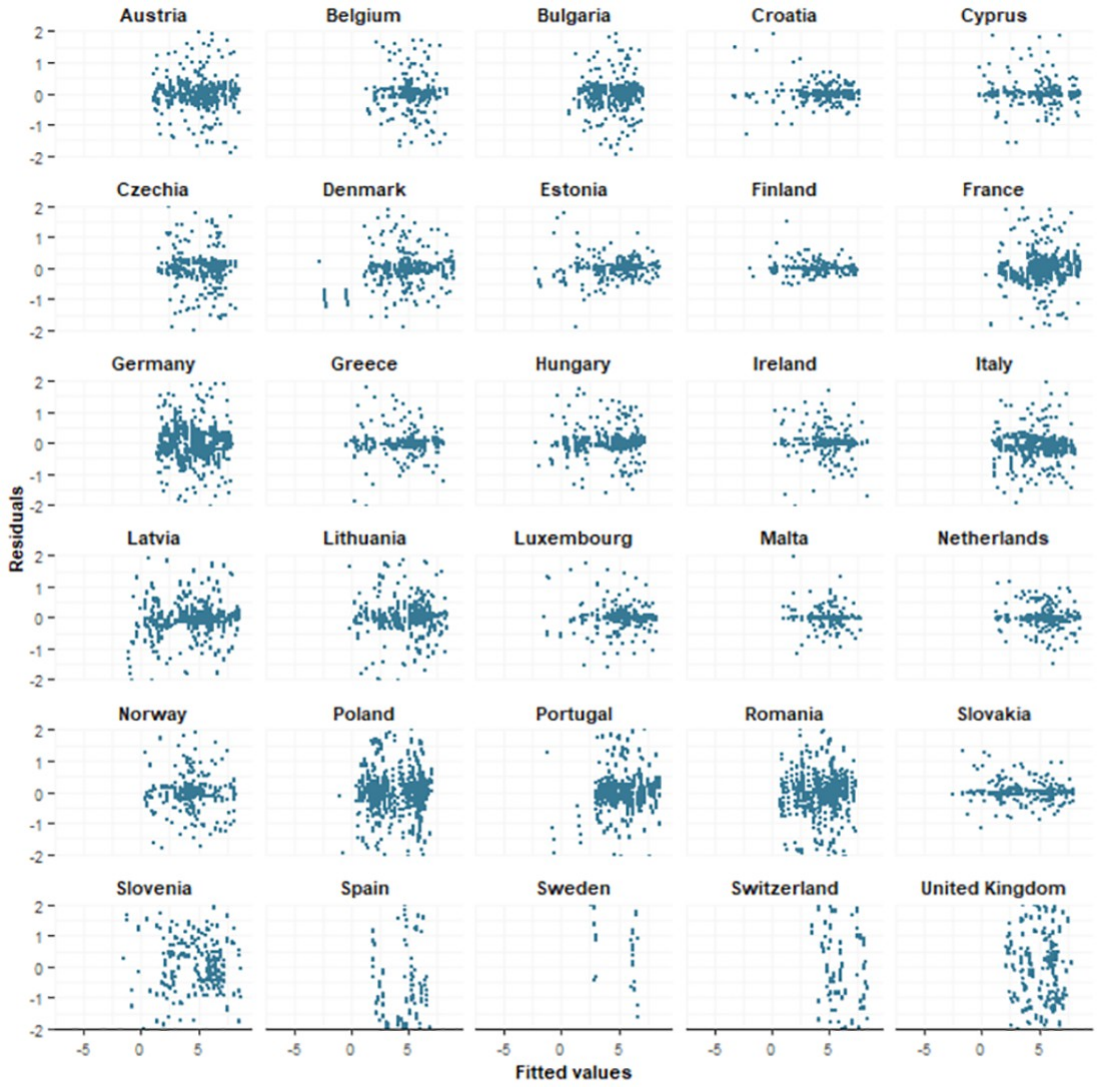
Standardized residuals positives. Graphical displays of the standardized residuals of the number of positive tests against the fitted values.

**Fig 5:**
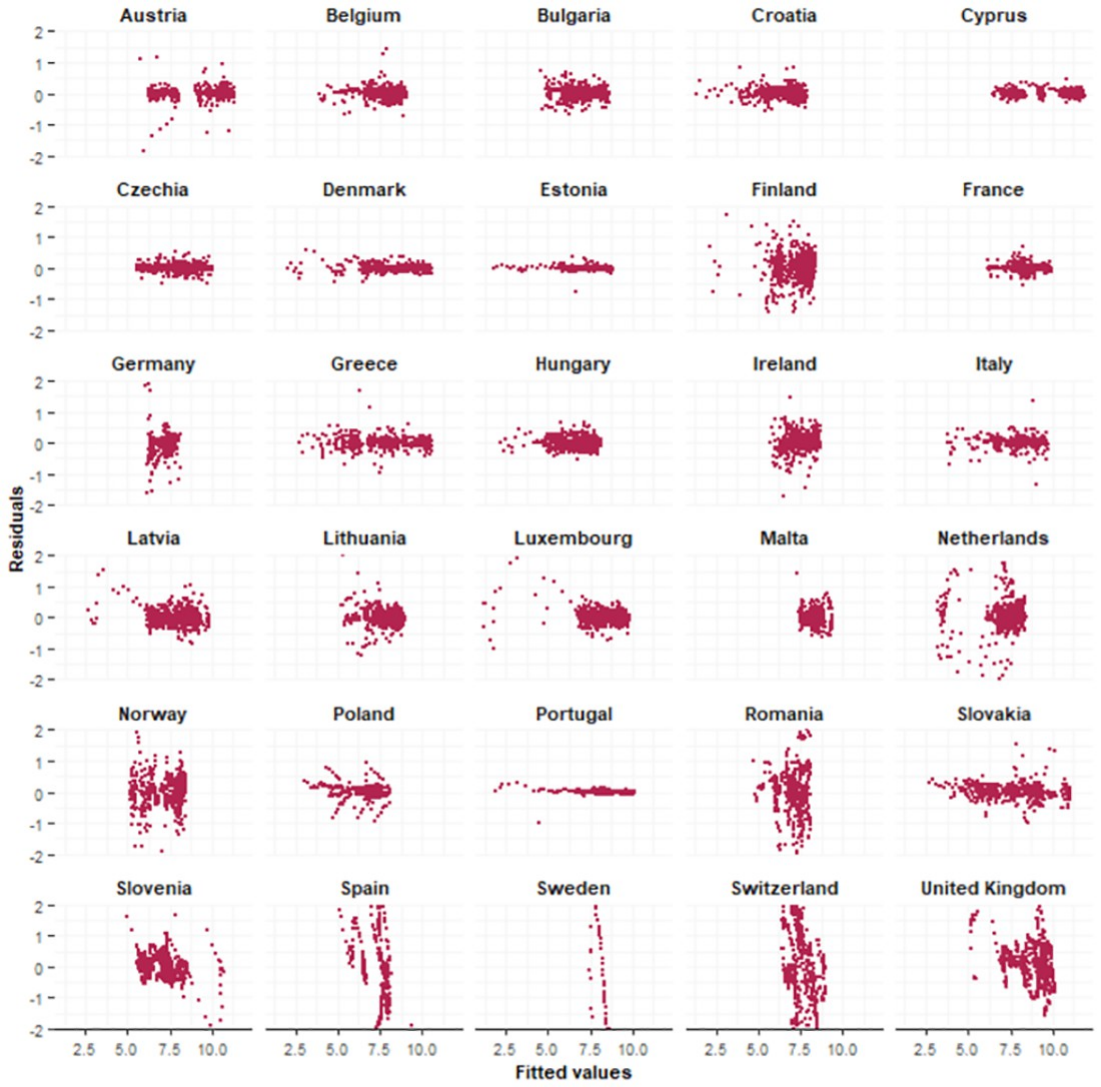
Standardized residuals negatives. Graphical displays of the standardized residuals of the number of negative tests against the fitted values.

We examined the standardized residuals by means of graphical plots. Standardized residuals were calculated as the difference between the observed values and the expected values, divided by the country-specific standard deviations of the error terms *ϵ*. In Figs 4 and 5, the standardized residuals were plotted against the fitted values. While there might be some heteroscedasticity present in the residuals for some countries, its impact is considered negligible due to the small magnitude of the standardized residuals.

To assess whether the added complexity of including the P-spline significantly improves the model’s fit, we compared the full model, which incorporates the P-splines, to a reduced model that only includes the linear trend. The DIC values demonstrate that the full model with P-splines (DIC: -97130) provides a better fit compared to the reduced model with only the linear trend (DIC: -85661). We have included the results of the model with only a linear trend in the Supplemental Information of the paper (S4 Table). Interestingly, in the reduced model, the correlations between the two random intercepts and between the two random slopes are still positive, but now, also the correlation between the random slopes became significant.

Additionally, to illustrate that the Gaussian model provides a better fit due its flexibility when compared to a negative binomial model for counts, a posterior predictive check was conducted by calculating a so-called Bayesian *p*-value for the Gaussian model described in (14) and a negative binomial model for the raw daily count data with the population size as an offset, an indicator variable for weekday and the smoothing splines. A Bayesian *p*-value close to 0.5 suggests a good fit, while low or high p-values suggest a lack of fit. The Bayesian *p*-value of the Gaussian model is equal to 0.5053, while the Bayesian *p*-value of the negative binomial model is 0.0837. This indicates that the Gaussian model indeed provides a better fit than the negative binomial model.

Lastly, the results of the sensitivity analysis, where we analyzed the daily number of positive and negative tests per 1,000,000 with the model described in (19), can be found in S5 Table. Similar conclusions can be drawn from the correlations between the random effects of the two outcomes. Positive correlations can be observed between both the random intercepts and random slopes, with a significant correlation found between the random intercepts.

Because the Omicron variant might have an impact on testing outcomes, we also fitted model 14 for the period excluding the Omicron wave. The correlations between the random effects together with the corresponding 95% credible intervals are given in Table 3. The correlation between the random intercepts, *δ*_0*i*,*p*_ and *δ*_0*i*,*n*_, was equal to 0.22, which is smaller compared to the correlation between the random intercepts considering the whole period. Moreover, this positive correlation is now non-significant. The correlation between the random slopes, *δ*_1*i*,*p*_ and *δ*_1*i*,*n*_, was estimated as 0.19, which is about the same as when the Omicron wave was included, and remains non-significant due to the wide credible interval.

**Table 3:** Correlations between the random intercepts and slopes excluding the Omicron wave.

	*δ*_0*i*,*p*_	*δ*_1*i*,*p*_	*δ*_0*i*,*n*_	*δ*_1*i*,*n*_
*δ*_0*i*,*p*_	1			
*δ*_1*i*,*p*_	-0.33 [-0.79; 0.14]	1		
*δ*_0*i*,*n*_	0.22 [-0.31; 0.72]	-0.23 [-0.67; 0.24]	1	
*δ*_1*i*,*n*_	-0.14 [-0.68; 0.40]	0.19 [-0.29; 0.66]	0.42 [-0.02; 0.81]	1

We also fitted the model separately for the Omicron wave (December 1, 2021 until June 23, 2022). The corresponding correlations and 95% credible intervals are given in Table 4. Both the random intercepts and the random slopes show a positive correlation. It is also interesting to note that these correlations are higher than the correlations when the Omicron wave is excluded and that these correlations are now significant. This indicates that countries with an on average higher number of positive tests, also have an on average higher number of negative tests.

**Table 4:** Correlations between the random intercepts and slopes only considering the Omicron wave.

	*δ*_0*i*,*p*_	*δ*_1*i*,*p*_	*δ*_0*i*,*n*_	*δ*_1*i*,*n*_
*δ*_0*i*,*p*_	1			
*δ*_1*i*,*p*_	0.61 [0.35; 0.84]	1		
*δ*_0*i*,*n*_	0.53 [0.25; 0.77]	0.51 [0.20; 0.78]	1	
*δ*_1*i*,*n*_	0.16 [-0.22; 0.54]	0.55 [0.25; 0.82]	0.38 [0.04; 0.71]	1

## 5 Discussion

The aim of this study was to provide insight into the relationship between the daily number of positive tests and the daily number of negative tests. For this purpose, we built a joint model using the random-effects approach. In this model, linear mixed models for the log-transformed 7-day rolling average number of new positive tests per 1,000,000 people and the log-transformed 7-day rolling average number of new negative tests per 1,000,000 people were built. To link both models, a multivariate normal distribution was imposed on the random intercepts and slopes. This is the first study which examines the association between the number of positive tests and the number of negative tests. However, we must emphasize that the association between the linear trends of the two outcomes may primarily capture the long-term correlation, but not the short-term correlation.

Since the longitudinal trajectories are nonlinear, a sufficiently flexible model was needed. Therefore, the main strength this study is the use of penalized splines. The linear mixed model representation is especially convenient for analyzing longitudinal data. Furthermore, we allowed the country-specific trajectories to be nonlinear functions as well by smoothing the country-specific trends. To the best of our knowledge, this is the first study that has used this methodology in a joint model for multivariate longitudinal data.

Another strength of the model is that we included an autoregressive structure for the error terms to account for autocorrelation. By doing so, we take into account serial correlation and we no longer make the assumption that the error terms are independent. Moreover, we allowed the residual variances and autocorrelation parameters to be country-specific to account for heteroscedasticity. This made the model even more flexible.

This study has some limitations. For instance, the number of tests is not purely a design variable, but is strongly influenced by the evolution of the epidemic itself. Growth of cases can be so rapid that it becomes increasingly difficult to scale up the testing efforts accordingly, which is reflected in an increasing positivity rate. At the same time, design and policy choices do influence the number of tests as well. For example, during periods of very high number of cases, some countries decided to stop testing the asymptomatic fraction.

Furthermore, different countries provide data using different definitions. First of all, for some countries, both PCR and antigen tests were included in the data, while other countries only included PCR tests. We also have no information on the proportion of antigen and PCR tests within the total number of positive and negative tests recorded in the data set. Therefore, we cannot differentiate between the types of tests conducted and their sensitivity and specificity. Given that the sensitivity and specificity for antigen tests and PCR tests is different [40–42], we cannot derive the rates of false positives and negatives and true positives and negatives, and we cannot provide insights into the sensitivity and specificity of these tests.

Some countries report the total number of tests performed, while other countries report the total number of individuals tested. There can be a dissimilarity between these two measures since the same person can be tested more than once [23]. Additionally, different countries have implemented distinct testing strategies, public health policies and reporting mechanisms, which may lead to heterogeneity between countries. Nevertheless, since the model incorporates country-specific random effects, including country-specific intercepts, linear trends, penalized splines, autocorrelation parameters and residual variances, the model provides the necessary flexibility to account for this heterogeneity between countries.

Next, we did not take into account spatial dependence, since contact patterns between countries were much smaller than contact patterns within a country. Thus, on the whole, contact patterns are very country-specific. Furthermore, each country employed different strategies and approaches to manage the pandemic and implemented its own set of policies and interventions, perhaps at different times. This heterogeneity in response strategies suggests that the impact of one country’s policies on another would be relatively small, which also may lead to limited spatial dependence.

Lastly, an autoregressive model of order one for the errors was implemented. However, we noticed that for some countries, there was still some autocorrelation left in the residuals. We examined this further by looking at the autocorrelation and partial autocorrelation functions of the residuals of the model without an AR(1) error structure. The partial autocorrelation functions suggested that, for some countries, an AR(1) error structure may not be sufficient, but an AR(2) error structure is needed. We tried to fit the model with an AR(2) error structure, but unfortunately this made the model too complex which led to convergence issues of the autocorrelation parameters. However, given that the residuals were quite small and based on Fig 3, we concluded that our model fits the data well.

The high and positive correlation between random intercepts suggests a strong association between countries with a high number of negative tests and a high number of positive tests. This indicates that countries exhibiting a higher number of negative tests also tend to have a higher number of positive tests. Consequently, our findings suggest that countries with higher mean testing capacities tend to exhibit higher numbers of both positive and negative tests. Similarly, a high correlation between random slopes would suggest a similar linear increase in numbers within countries. This implies that countries experiencing an upward trend in the number of negative tests also tend to exhibit a similar pattern in the increase of positive tests over time. This may indicate that countries that experienced a linear increase in their testing capacities during the study period, also exhibited a corresponding linear increase in both the number of positive and negative tests. As a consequence, an increase in the number of daily cases cannot be fully explained by an increase in the daily number of total tests conducted, since the number of negative tests also increases when the number of positive tests increases. However, only the correlation between the random intercepts was significant, while the correlation between the random slopes was non-significant due to a wide credible interval. This may indicate that various other factors influence both the number of positive and negative tests, such as changes in disease transmission or testing patterns. When the Omicron wave was excluded, the correlation between the random intercepts became also non-significant, but was still positive. When we performed the analysis on the Omicron wave only, both the correlation between the random intercepts and the correlation between the random slopes were significant.

## Supporting information

S1 Table Summary statistics positive tests. Summary statistics of the number of positive tests for each country separately.
(PDF)

S2 Table Summary statistics negative tests. Summary statistics of the number of negative tests for each country separately.
(PDF)

S3 Table Country-specific parameters. Estimated country-specific autocorrelation parameters and residual variances.
(PDF)

S4 Table Correlations of the reduced model. Correlations between the random intercepts and slopes in the model with only a linear trend.
(PDF)

S5 Table Correlations of the model for the daily data. Correlations between the random intercepts and slopes in the model for the daily number of positive and negative tests per 1,000,000.
(PDF)

S1 Fig Number of positive COVID-19 tests. Graphical displays of the 7-day rolling average number of positive COVID-19 tests.
(TIFF)

S2 Fig Number of negative COVID-19 tests. Graphical displays of the 7-day rolling average number of negative COVID-19 tests.
(TIFF)

S3 Fig Country-specific parameters. Graphical displays of the country-specific autocorrelation parameters and residual variances.
(TIFF)

S4 Fig Estimated and observed trajectories. The estimated log-transformed 7-day rolling average number of positive (blue) and negative (red) tests for each country (solid lines) and the observed log-transformed 7-day rolling average number of positive (blue) and negative (red) tests for each country (dashed lines).
(TIFF)

S5 Fig Positivity rate. Graphical display of the positivity rate.
(TIFF)
