# Supplementary material for: A joint penalized spline smoothing model for the number of positive and negative COVID-19 tests
Source: PLoS One. 2024 May 6;19(5):e0303254. doi: 10.1371/journal.pone.0303254 (PMC11073685; doi:10.1371/journal.pone.0303254)
Supplement: S1 Table — Summary statistics of the number of positive tests for each country separately. (PDF) [file pone.0303254.s001.pdf]

	location	Mean	Median	Max	Min
1	Austria	464.66	183.29	5099.42	0.00
2	Belgium	284.79	127.33	4122.34	0.00
3	Bulgaria	133.83	32.10	1302.43	0.00
4	Croatia	221.97	75.68	2110.30	0.00
5	Cyprus	519.24	239.95	5083.82	0.00
6	Czechia	309.72	66.20	3901.17	0.00
7	Denmark	398.82	88.67	7833.39	0.00
8	Estonia	314.25	66.15	5174.39	0.00
9	Finland	184.88	46.02	1584.32	0.00
10	France	495.42	245.98	5342.87	0.00
11	Germany	376.06	152.66	2722.62	0.00
12	Greece	369.87	176.93	3319.65	0.08
13	Hungary	152.83	32.31	1554.91	0.00
14	Ireland	235.84	63.31	4197.26	0.00
15	Italy	309.09	101.23	3068.64	0.73
16	Latvia	360.86	79.20	5289.02	0.00
17	Lithuania	338.95	116.88	4587.02	0.00
18	Luxembourg	412.56	188.17	4061.80	0.22
19	Malta	155.49	39.91	2174.63	0.00
20	Netherlands	335.82	67.34	5094.03	0.00
21	Norway	189.40	32.23	3727.21	0.00
22	Poland	114.14	18.13	1198.50	0.00
23	Portugal	375.27	63.15	5637.39	0.01
24	Romania	122.45	39.80	1518.64	0.03
25	Slovakia	227.25	20.55	3140.58	0.00
26	Slovenia	436.85	163.89	6659.93	0.00
27	Spain	239.77	102.42	2873.16	0.00
28	Sweden	178.12	51.84	3805.76	0.18
29	Switzerland	347.95	108.20	4066.29	0.73
30	United Kingdom	252.02	67.40	2793.98	0.07
