# Supplementary material for: A joint penalized spline smoothing model for the number of positive and negative COVID-19 tests
Source: PLoS One. 2024 May 6;19(5):e0303254. doi: 10.1371/journal.pone.0303254 (PMC11073685; doi:10.1371/journal.pone.0303254)
Supplement: S2 Table — Summary statistics of the number of negative tests for each country separately. (PDF) [file pone.0303254.s002.pdf]

	location	Mean	Median	Max	Min
1	Austria	25704.82	26856.57	87036.61	6.89
2	Belgium	3110.17	3164.90	8825.61	50.14
3	Bulgaria	1617.02	1364.23	5480.15	89.39
4	Croatia	1109.98	1169.45	2660.51	3.79
5	Cyprus	43450.28	46681.20	143501.79	658.85
6	Czechia	6430.46	4837.48	22631.65	258.16
7	Denmark	12445.55	10187.10	38247.47	7.93
8	Estonia	2550.64	2662.17	6721.37	5.89
9	Finland	2129.81	2291.87	4683.16	7.11
10	France	4827.59	4088.93	20169.43	458.93
11	Germany	1535.45	1575.67	3377.01	370.85
12	Greece	9240.83	3992.07	39080.01	15.90
13	Hungary	1183.36	1087.44	3496.62	8.90
14	Ireland	2626.90	2601.54	6519.81	39.74
15	Italy	4133.64	3366.04	16128.72	39.46
16	Latvia	4087.12	3739.23	19031.25	15.00
17	Lithuania	3321.38	2776.71	7653.89	182.49
18	Luxembourg	7462.72	6695.82	18504.67	2.78
19	Malta	4729.50	4476.97	12202.48	1678.25
20	Netherlands	1739.74	1534.19	5397.29	0.00
21	Norway	2233.67	2557.58	5448.49	0.00
22	Poland	992.41	969.82	3365.71	21.65
23	Portugal	4534.71	3273.60	24085.42	6.62
24	Romania	1340.95	1245.35	3620.33	10.12
25	Slovakia	10921.64	4536.33	63518.14	14.92
26	Slovenia	2401.65	1377.91	43513.53	13.00
27	Spain	2070.44	2019.65	6619.32	164.82
28	Sweden	2131.19	1974.93	6002.17	0.00
29	Switzerland	2693.36	2612.31	7867.34	372.89
30	United Kingdom	9232.52	9293.32	24886.02	171.95
