# Supplementary material for: A joint penalized spline smoothing model for the number of positive and negative COVID-19 tests
Source: PLoS One. 2024 May 6;19(5):e0303254. doi: 10.1371/journal.pone.0303254 (PMC11073685; doi:10.1371/journal.pone.0303254)
Supplement: S3 Table — Estimated country-specific autocorrelation parameters and residual variances. (PDF) [file pone.0303254.s003.pdf]

	Location	ρ_p	ρ_n	$\sigma^2_{\omega_p}$	$\sigma^2_{\omega_n}$
1	Austria	0.8513	0.9096	0.0128	0.0514
2	Belgium	0.9307	0.9897	0.0159	0.0022
3	Bulgaria	0.8659	0.9871	0.0139	0.0042
4	Croatia	0.9740	0.9910	0.0389	0.0042
5	Cyprus	0.9619	0.9970	0.0306	0.0021
6	Czechia	0.9001	0.9948	0.0139	0.0021
7	Denmark	0.8656	0.9966	0.0303	0.0019
8	Estonia	0.8867	0.9989	0.1097	0.0067
9	Finland	0.9897	0.8779	0.0214	0.0021
10	France	0.8012	0.9958	0.0145	0.0025
11	Germany	0.7460	0.9429	0.0082	0.0026
12	Greece	0.9405	0.9879	0.0195	0.0055
13	Hungary	0.9077	0.9895	0.0263	0.0033
14	Ireland	0.9630	0.7562	0.0220	0.0283
15	Italy	0.8650	0.9907	0.0140	0.0013
16	Latvia	0.6951	0.9927	0.0473	0.0033
17	Lithuania	0.8403	0.9908	0.0217	0.0038
18	Luxembourg	0.9407	0.9925	0.0380	0.0081
19	Malta	0.9794	0.9932	0.0378	0.0007
20	Netherlands	0.9722	0.4977	0.0172	0.0978
21	Norway	0.9371	0.5794	0.0121	0.0419
22	Poland	0.6268	0.9966	0.0136	0.0017
23	Portugal	0.7364	0.9998	0.0165	0.0021
24	Romania	0.5960	0.8214	0.0122	0.0016
25	Slovakia	0.9726	0.9934	0.0315	0.0045
26	Slovenia	0.8811	0.9935	0.0786	0.0069
27	Spain	0.8163	0.9923	0.0109	0.0015
28	Sweden	0.8000	0.9110	0.0124	0.0099
29	Switzerland	0.9079	0.9916	0.0131	0.0022
30	United Kingdom	0.9165	0.9947	0.0084	0.0011
