# Supplementary material for: A joint penalized spline smoothing model for the number of positive and negative COVID-19 tests
Source: PLoS One. 2024 May 6;19(5):e0303254. doi: 10.1371/journal.pone.0303254 (PMC11073685; doi:10.1371/journal.pone.0303254)
Supplement: S4 Table — Correlations between the random intercepts and slopes in the model with only a linear trend. (PDF) [file pone.0303254.s004.pdf]

	$\delta_{0i,p}$	$\delta_{1i,p}$	$\delta_{0i,n}$	$\delta_{1i,n}$
$\delta_{0i,p}$	1			
$\delta_{1i,p}$	-0.27<br>[-0.75; 0.23]	1		
$\delta_{0i,n}$	0.69<br>[0.46; 0.88]	0.06<br>[-0.39; 0.49]	1	
$\delta_{1i,n}$	0.10<br>[-0.29; 0.50]	0.43<br>[0.07; 0.76]	0.44<br>[0.11; 0.74]	1
