# Supplementary material for: A joint penalized spline smoothing model for the number of positive and negative COVID-19 tests
Source: PLoS One. 2024 May 6;19(5):e0303254. doi: 10.1371/journal.pone.0303254 (PMC11073685; doi:10.1371/journal.pone.0303254)
Supplement: S5 Table — Correlations between the random intercepts and slopes in the model for the daily number of positive and negative tests per 1,000,000. (PDF) [file pone.0303254.s005.pdf]

	$\delta_{0i,p}$	$\delta_{1i,p}$	$\delta_{0i,n}$	$\delta_{1i,n}$
$\delta_{0i,p}$	1			
$\delta_{1i,p}$	0.25<br>[-0.03; 0.53]	1		
$\delta_{0i,n}$	0.45<br>[0.17; 0.73]	0.18<br>[-0.10; 0.47]	1	
$\delta_{1i,n}$	0.07<br>[-0.20; 0.35]	0.34<br>[0.05; 0.62]	0.63<br>[0.34; 0.82]	1
