# Supplementary figures and images for: A joint penalized spline smoothing model for the number of positive and negative COVID-19 tests
Source: PLoS One. 2024 May 6;19(5):e0303254. doi: 10.1371/journal.pone.0303254 (PMC11073685; doi:10.1371/journal.pone.0303254)

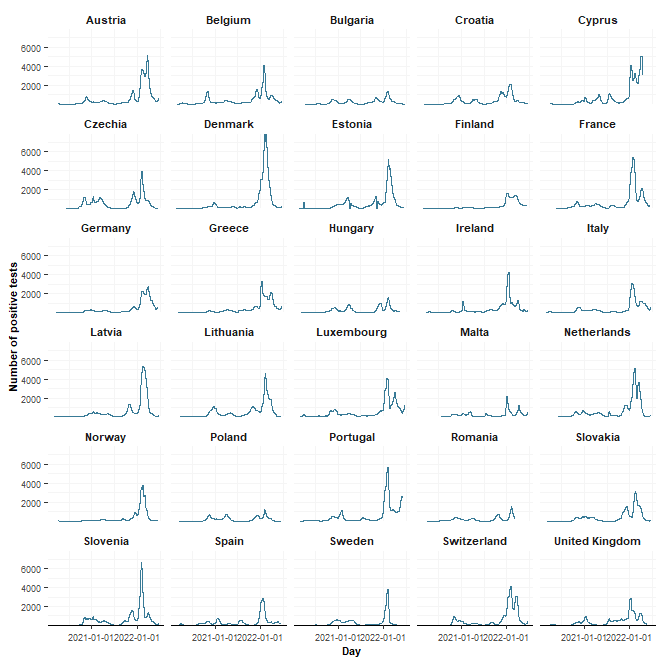

Supplement: S1 Fig — Graphical displays of the 7-day rolling average number of positive COVID-19 tests. (TIFF) [file pone.0303254.s006.tiff]

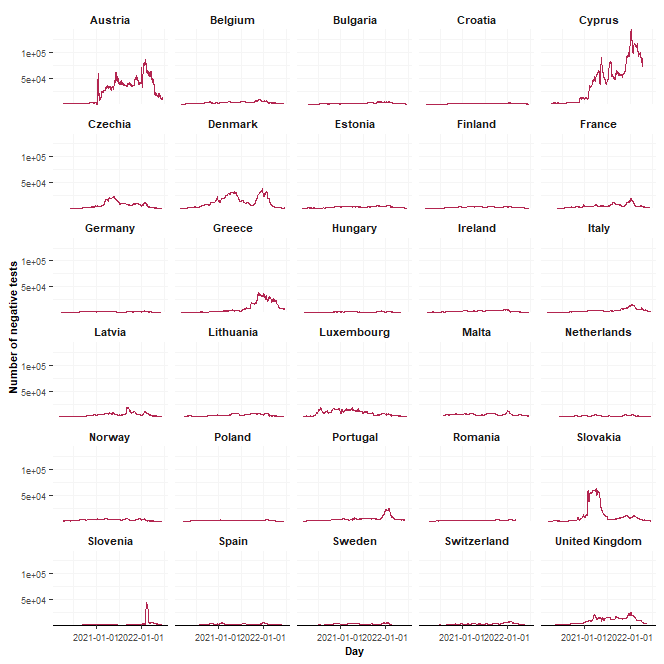

Supplement: S2 Fig — Graphical displays of the 7-day rolling average number of negative COVID-19 tests. (TIFF) [file pone.0303254.s007.tiff]

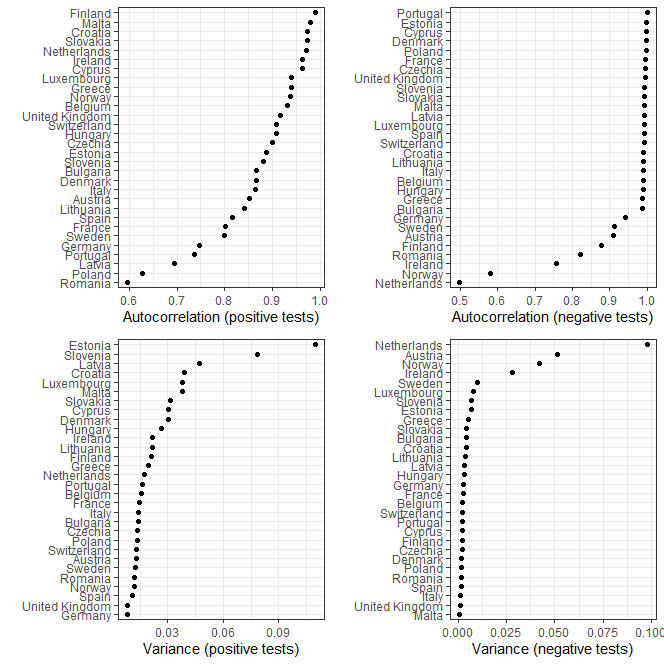

Supplement: S3 Fig — Graphical displays of the country-specific autocorrelation parameters and residual variances. (TIFF) [file pone.0303254.s008.tiff]

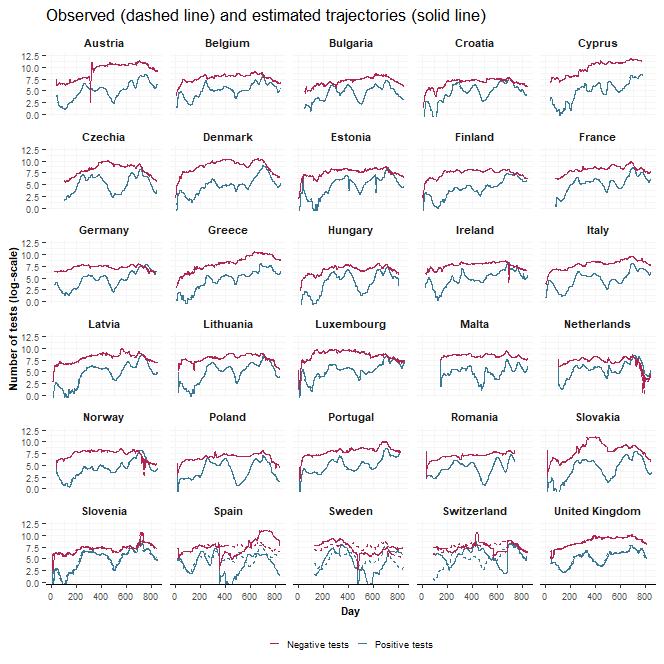

Supplement: S4 Fig — The estimated log-transformed 7-day rolling average number of positive (blue) and negative (red) tests for each country (solid lines) and the observed log-transformed 7-day rolling average number of positive (blue) and negative (red) tests for each country (dashed lines). (TIFF) [file pone.0303254.s009.tiff]

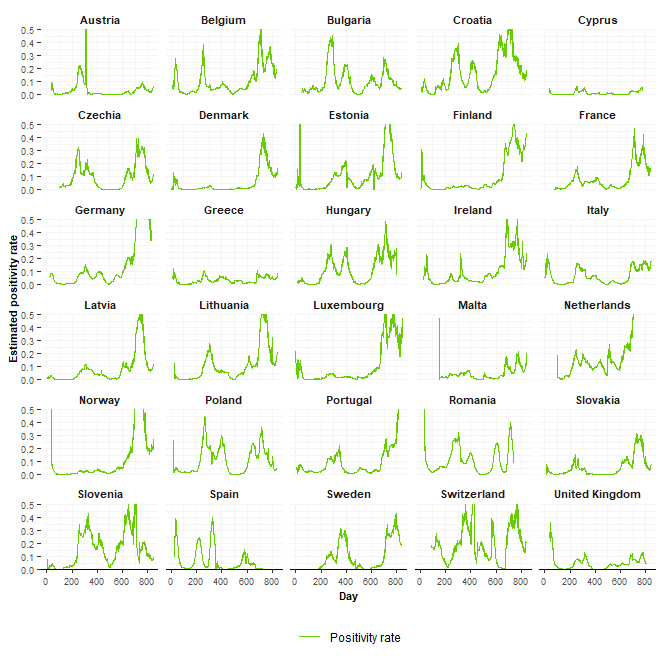

Supplement: S5 Fig — Graphical display of the positivity rate. (TIFF) [file pone.0303254.s010.tiff]
